# Impact of Age-Dependent Adventitia Inflammation on Structural Alteration of Abdominal Aorta in Hyperlipidemic Mice

**DOI:** 10.1371/journal.pone.0105739

**Published:** 2014-08-25

**Authors:** Sumiharu Sakamoto, Toshihiro Tsuruda, Kinta Hatakeyama, Takuroh Imamura, Yujiro Asada, Kazuo Kitamura

**Affiliations:** 1 Department of Internal Medicine, Circulatory and Body Fluid Regulation, Faculty of Medicine, University of Miyazaki, Miyazaki, Japan; 2 Department of Pathology, Faculty of Medicine, University of Miyazaki, Miyazaki, Japan; 3 Department of Internal Medicine, Koga General Hospital, Miyazaki, Japan; University of Pittsburgh School of Medicine, United States of America

## Abstract

**Background:**

The adventitia is suggested to contribute to vascular remodeling; however, the site-selective inflammatory responses in association with the development of atherosclerosis remain to be elucidated.

**Methods and Results:**

Wild-type or apolipoprotein E knockout male C57BL/6J background mice were fed standard chow for 16, 32, and 52 weeks, and the morphology of the aortic arch, descending aorta, and abdominal aorta was compared. Atheromatous plaque formation progressed with age, particularly in the aortic arch and abdominal aorta but not in the descending aorta. In addition, we found that the numbers of macrophages, T-lymphocytes, and microvessels, assessed by anti-F4/80, CD3, and CD31 antibodies, were higher in the adventitia of the abdominal aorta at 52 weeks. These numbers were positively correlated with plaque formation, but negatively correlated with elastin content, resulting in the enlargement of the total vessel area. In aortic tissues, interleukin-6 levels increased in the atheromatous plaque with age, whereas the level of regulated on activation, normal T cell expressed and secreted (RANTES) increased with age, and compared with other sites, it was particularly distributed in inflammatory cells in the adventitia of the abdominal aorta.

**Conclusion:**

This study suggests that adventitial inflammation contributes to the age-dependent structural alterations, and that the activation/inactivation of cytokines/chemokines is involved in the process.

## Introduction

Atherosclerosis is a hallmark of cardiovascular diseases such as stroke and myocardial infarction, and inflammation of the arterial wall is involved in the pathology [1], [2], [3]. Aging, dyslipidemia, hypertension, diabetes mellitus and smoking lead to inflammatory responses and progression of atherosclerosis [2]. Injured endothelial cells initiate the incorporation of oxidized low-density lipoprotein cholesterol and stimulate the proliferation/migration of smooth muscle cells in the arterial wall [1], [4]. These actions are mediated/activated by various adhesion molecules and cytokines [5]. The lamina adventitia is defined as the area surrounding the vasculature, from outside the external elastic lamina (EEL) to the edge of the perivascular adipose tissues. The adventitia contains terminal nerve fibers, fibroblasts, collagen and some inflammatory cells [6], [7]. Recent studies have suggested that the adventitial layer not only supports the structure of the arterial wall against blood pressure, but also contributes to neointimal formation and destabilization of atheromatous plaques [8], [9], [10], [11], [12], [13].

Mice homozygous for apolipoprotein E (apo E^−/−^) exhibit a marked increase in the plasma level of total cholesterol, accompanied by the progression of atheromatous plaque with age, which resembles human atherosclerosis [14], [15]. Thus, this model is useful to examine the mechanism underlying atherosclerosis and atherosclerosis-based cardiovascular diseases. However, few studies have addressed the site-selective inflammatory responses in the adventitia in association with the development of atherosclerosis. Therefore, we tested whether adventitial inflammation is associated with structural alterations of the aorta in age- and site-specific manners in a mouse model of hyperlipidemia.

## Materials and Methods

This study was performed in accordance with the Animal Welfare Act, with approval of the University of Miyazaki Institutional Animal Care and Use Committee (#2013-505) and of University of Miyazaki for genetic modification (#151-3 and #230). This study also conformed to the *Guide for the Care and Use of Laboratory Animals* published by the US National Institutes of Health (8^th^ edition, 2011).

### Animal experiments

A pair of male and female apo E^−/−^ knockout mice (B6.129P2-*Apoe^tm1Unc^*/J, background strain C57BL/6J) were purchased from Jackson Laboratory (Bar Harbor, Maine, USA) and bred at our institution. Wild-type male mice (C57BL/6J) were purchased from Charles River (Yokohama, Japan). They were housed in a temperature- and light-controlled room (25°C±1°C; 12/12-h light/dark cycle) with free access to normal chow and water until the end of the experiment. At 16, 32, and 52 weeks of age, the mice were anesthetized by injecting pentobarbital sodium (80 mg/kg) intraperitoneally, and the aortic tree was harvested from the ascending aorta to the bifurcation of the common iliac arteries. They were perfused with 4% paraformaldehyde or phosphate-buffered saline and stored in 4% paraformaldehyde or frozen in liquid nitrogen. Apo E^−/−^ mice (16 weeks old, n = 5; 32 weeks old, n = 7; 52 weeks old, n = 9) and wild-type mice (16 weeks old, n = 9; 32 weeks old, n = 5; 52 weeks old, n = 10) were used for histological assessment. In addition, aortic samples were prepared to measure the tissue concentration of cytokines/chemokines (wild-type mice: 16 weeks old, n = 3; 32 weeks old, n = 3; 52 weeks old, n = 4; apo E^−/−^ mice: 16 weeks old, n = 3; 32 weeks old, n = 3; 52 weeks old, n = 4).

### Blood pressure and heart rate

Blood pressure was measured using a noninvasive computerized tail-cuff system (BP98A Softron, Tokyo, Japan) while the animal was conscious. The mean blood pressure and heart rate were determined from at least 3 successful measurements after 2 days of training.

### Blood samples

Blood samples were collected from the left ventricle under anesthesia. The samples were mixed with 10 µl of heparin and centrifuged at 3,000 rpm (4°C; 10 min). The plasma was stored at −80°C until use. The total cholesterol was analyzed by an enzyme method using a dry chemistry analyzer (FUJI DRI-CHEM 3500, FUJIFILM, Tokyo, Japan) and FUJI DRI-CHEM slides (TCHO-P3, FUJIFILM).

### Histology

The collagen and elastin contents of the aortic wall were evaluated by Sirius red and Victoria blue staining respectively [16], [17]. In brief, 3-µm-thick tissue sections were deparaffinized, rehydrated, and incubated for 10 min with 0.1% Sirius red stain in saturated picric acid. For evaluating the elastin content of the vessel wall, tissue sections were stained with Victoria blue stain.

### Immunohistochemistry

Immunohistochemistry was performed as described previously [16]. In brief, 3-µm-thick sections of the aorta, fixed in 4% paraformaldehyde, were deparaffinized with xylene and graded alcohol from 100% to 70% and autoclaved at 121°C for 15 min in 10 mmol/l citrate buffer (pH 6.0). Following this, section slides were immersed in 3% H_2_O_2_ for 20 min to block endogenous peroxidase activity, followed by 10 min in Protein Block Serum-Free Ready-to-Use solution (DakoCytomation Japan, Kyoto, Japan) to reduce the non-specific background. The slides reacted with specific polyclonal antibodies against CD3 (1∶100, ab5690; Abcam, Cambridge, UK), CD31 (ab28365; Abcam), interleukin (IL)-6 (1∶400, ab6672; Abcam), and regulated on activation, normal T cell expressed and secreted (RANTES) (1∶50, NBP1-19769, Novus, Littleton, CO, USA) or monoclonal antibodies against F4/80 (1∶10,000, CL8940AP, Cedarlane, Hornby, ON, Canada) at 4°C overnight. Following this, they reacted with peroxidase-based EnVision+ (DakoCytomation Japan) for 30 min at room temperature, and were developed in 0.05% 3,3′-diaminobenzidine containing hydrogen peroxide. All sections were counterstained with hematoxylin and eosin. A catalyzed signal amplification system (CSA-Dako–Cytomation) was used to enhance the detection of F4/80 antigen, according the to manufacturer's instructions.

### Morphological assessment

A single observer (S.S.) evaluated the morphology of the harvested aorta in a blind manner. The atheromatous plaque area, lumen area, and total vessel area circumscribed by EEL were estimated by computerized measurements (WinRoof, version 5.6, Mitani Co., Tokyo, Japan). The media area was calculated as the EEL area minus the internal elastic lamina (IEL) area. The numbers of macrophages (F4/80), T-lymphocytes (CD3), and microvessels (CD31) located in the adventitia were counted in the aortic arch, descending aorta, and abdominal aorta (×400 magnitude). Microvessels were identified as circular structures less than or equal to 50 µm in diameter delimited by CD31-positive cells. The average number of these types of cells or microvessels was determined individually or corrected for the area of the adventitia. The amount of collagen deposition scanned under polarized light and the amount of elastic fibers positive for Victoria blue staining were determined by computerized measurements (WinRoof, version 5.6, Mitani Co.). The diameter of >100 perivascular adipocytes per cross-section was measured using WinRoof (version 6.5, Mitani Co.) and then averaged. In the case of adipocyte sections in an oblique orientation, the smallest axis was used as the diameter.

### Tissue protein concentration

The aortae were divided into 3 segments (aortic arch, descending aorta, and abdominal aorta) and thawed on ice in lysis buffer containing 1% Triton X, 50 mM 4-(2-hydroxyethyl)-1-piperazineethanesulfonic acid (HEPES), 50 mM NaCl, and a protease inhibitor cocktail (Cat. No. 04 693 159 001; Roche diagnostics GmbH, Mannheim, Germany). Samples were then centrifuged at 12,360 rpm (10 min; 4°C), and the supernatants were stored at −80°C. The protein concentration was determined by the bicinchoninic acid assay using a spectrophotometer (U-2000, HITACHI, Tokyo, Japan).

### Cytokine/chemokine analysis

Tissue concentrations of IL-6, IL-10, and RANTES were measured using a multiplexed bead-based immunoassay kit (Bio-Rad, Hercules, CA, USA), according to the manufacturer's instructions. Standards and samples were processed using the Bio-Plex Protein Array System (Bio-Rad) and analyzed with Bio-Plex Manager software, version 5.0 (Bio-Rad).

### Statistical analysis

All data are expressed as mean ± standard error of the mean (SEM). Mann–Whitney tests were used to compare continuous variables between wild-type and apo E^−/−^ mice. Relationships between continuous variables were determined by Spearman's rank test. The differences were accepted as statistically significant when p<0.05. All tests were conducted using PRISM 5.01 (GraphPad Software, San Diego, CA, USA).

## Results

### Body weight, systolic blood pressure, heart rate, and plasma cholesterol

Table 1 shows the impact of age on the characteristics of the apo E^−/−^ mice and wild-type mice used in this study. Body weight was significantly higher in apo E^−/−^ mice than in wild-type mice at 16 weeks but significantly lower at 52 weeks. The plasma cholesterol level was significantly higher in apo E^−/−^ mice than in wild-type mice throughout the experimental period. The systolic blood pressure and heart rate did not differ between the 2 groups.

**Table 1:** Impact of hyperlipidemia on the characteristics of the mice.

	16-week-old mice	32-week-old mice	52-week-old mice
**Wild-type**			
**BW** (g)	24.9±0.4 (12)	30.8±0.4 (8)	38.3±1 (14)
**SBP** (mmHg)	99±2 (12)	99±1 (8)	96±3 (14)
**HR** (beats/min)	652±6 (12)	655±10 (8)	683±13 (14)
**T-Cho** (mg/dl)	56±2 (11)	54±3 (8)	61±4 (14)
**Apo E^−/−^**			
**BW** (g)	28.0±0.8 * (8)	30.6±0.7 (10)	33.9±0.7 ** (13)
**SBP** (mmHg)	102±2 (8)	99±2 (10)	97±2 (13)
**HR** (beats/min)	648±28 (8)	679±8 (10)	667±10 (13)
**T-Cho** (mg/dl)	687±81 *** (5)	707±70 *** (9)	702±55 *** (12)

Data are expressed as mean ± SEM (Parentheses indicate the number examined).

*P<0.05, **P<0.001, ***P<0.0001 vs. wild-type.

BW, body weight; SBP, systolic blood pressure; HR, heart rate; T-Cho, total cholesterol.

Apo E^−/−^, apo E knockout mice.

### Atheromatous plaque, elastin, and collagen in aortic wall

**Figure 1:**
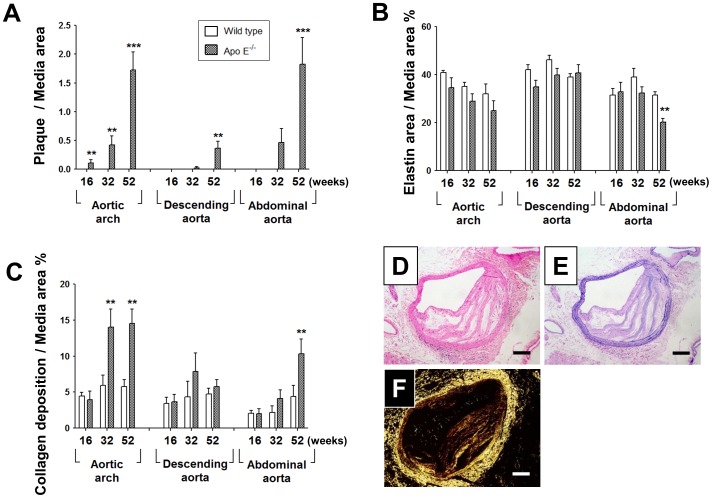
Impact of age on the structure of the aorta in wild-type and apo E^−/−^ mice. (**A–C**) Atheromatous plaque area (**A**), elastin area (**B**), and collagen deposition (**C**) at each age and aorta region in apo E^−/−^ and wild-type mice. (**D–F**) Representative images of hematoxylin–eosin (**D**), Victoria blue (**E**), and picrosirius red (**F**) staining in the abdominal aorta of apo E^–/–^ mice at 52 weeks. Data are expressed as mean ± SEM [16 weeks: wild-type (n = 9), apo E^–/–^ mice (n = 5–6); 32 weeks: wild-type (n = 5–7), apo E^–/–^ mice (n = 6–7); 52 weeks: wild-type (n = 10), apo E^–/–-^ mice (n = 9–11)]. **p<0.01, ***p<0.001 vs. wild-type mice. Scale bar, 100 µm.

The structure of the aorta was extensively modified by hyperlipidemia. In apo E^−/−^ mice, atheromatous plaque formation progressed with age in the aortic arch and abdominal aorta, but to a lesser extent in the descending aorta (Figure 1A). The elastin content was preserved in the aortic arch and descending aorta throughout the experimental period. However, compared with wild-type mice, it was significantly reduced in the abdominal aorta of 52-week-old apo E^−/−^ mice (Figure 1B). Compared with wild-type mice, collagen deposition was significantly higher in the aortic arch at 32 and 52 weeks in the abdominal aorta at 52-weeks in apo E^−/−^ mice (Figure 1C). Figure 1D–F shows representative images of hematoxylin–eosin staining (Figure 1D), Victoria blue staining (Figure 1E), and picrosirius red staining under polarized light (Figure 1F) in the abdominal aorta of 52-week-old apo E^−/−^ mice.

### Adventitial inflammatory cells and microvessels, and perivascular adipose tissues

**Figure 2:**
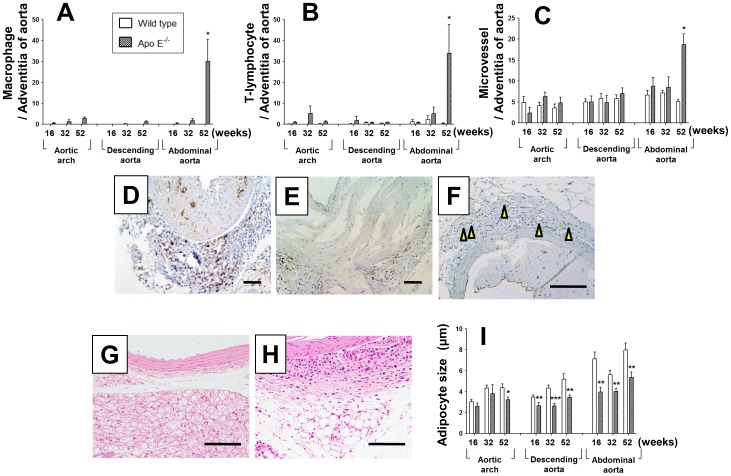
Impact of age on adventitia inflammatory cells, microvessels, and perivascular adipocytes. (**A–C**) Numbers of F4/80-positive macrophages (**A**), CD3-positive T-lymphocytes (**B**), and CD31-positive microvessels (**C**) in the adventitia of the aorta at each age and region in apo E^−/−^ and wild-type mice. (**D–F**) Representative images of F4/80 (**D**), CD3 (**E**), and CD31 (**F**) immunostaining in the abdominal aorta of apo E^−/−^ mice at 52 weeks. Arrows indicate CD31-positive microvessels in the adventitia. (**G, H**) Representative hematoxylin–eosin staining of perivascular adipose tissue in the descending aorta (**G**) and abdominal aorta (**H**) of 52-week-old apo E^−/−^ mice. (**I**) Diameter of adipocytes surrounding the aortic arch, descending aorta, and abdominal aorta. Data are expressed as mean ± SEM [16 weeks: wild-type (n = 9), apo E^−/−^ mice (n = 5–6); 32 weeks: wild-type (n = 5–7), apo E^−/−^ mice (n = 6–7); 52 weeks: wild-type (n = 10), apo E^−/−^ mice (n = 9–11)]. *p<0.05, **p<0.01 vs. wild-type mice. Scale bar, 100 µm.

The numbers of macrophages and T-lymphocytes infiltrated in the adventitial layer were markedly elevated in the abdominal aorta of 52-week-old apo E^–/–^ mice, whereas few were observed in the aortic arch or descending aorta throughout the experimental period (Figure 2A and B). Microvessels were significantly higher in the abdominal aorta of 52-week-old apo E^–/–^ mice than that of wild-type mice (Figure 2C). Figure 2D–F shows representative images of F4/80-positive macrophages (Figure 2D), CD3-positive T-lymphocytes (Figure 2E), and CD31-positive microvessels (Figure 2F) in the abdominal aorta of 52-week-old apo E^–/–^ mice. Plurilocular adipocytes were observed in the aortic arch and descending aorta (Figure 2G), whereas unilocular adipocytes were predominantly distributed in the abdominal aorta (Figure 2H). The size of the perivascular adipocytes increased with age in the descending and abdominal aorta of wild-type mice; however, the size was significantly lesser in apo E^–/–^ mice (Figure 2I).

### Relationship between structural changes and adventitial inflammation in the abdominal aorta

**Figure 3:**
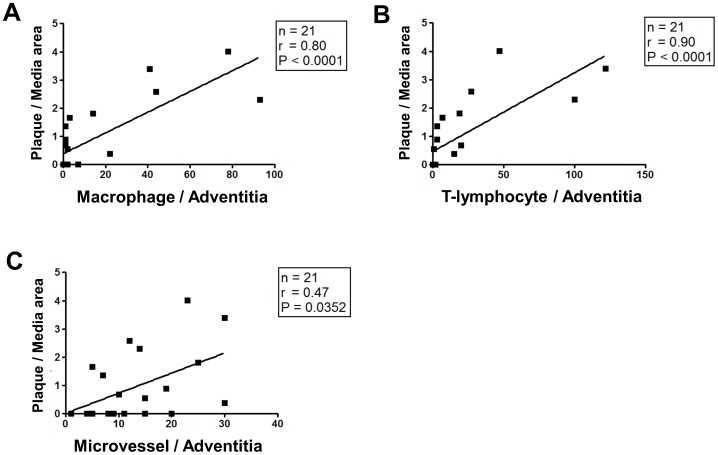
Relationship between plaque area and adventitial inflammation in the abdominal aorta. (**A–C**) Liner regression between plaque/media area and the number of [macrophages (**A**), T-lymphocytes (**B**), and microvessels (**C**)] in the adventitia of 52-week-old apo E^−/−^ mice.

**Figure 4:**
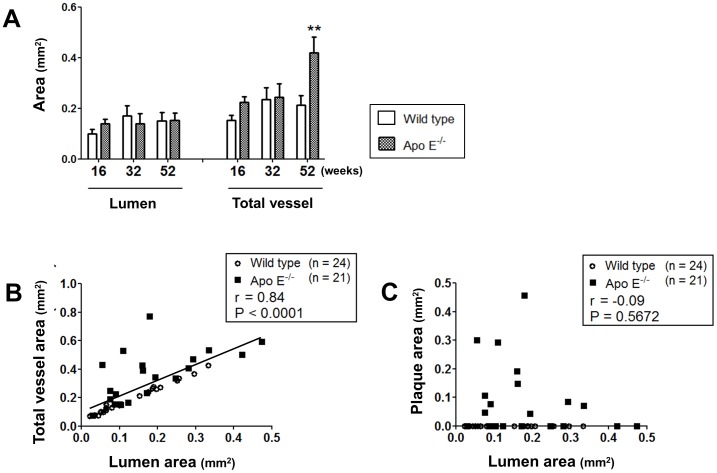
Relationship between lumen area and total vessel area in the abdominal aorta. (**A**) Impact of age on lumen and total vessel area in the abdominal aorta of apo E^−/−^ and wild-type mice. Data are expressed as mean ± SEM [16 weeks: wild-type (n = 9), apo E^−/−^ mice (n = 5); 32 weeks: wild-type (n = 5), apo E^−/−^ mice (n = 7); 52 weeks: wild-type (n = 10), apo E^−/−^ mice (n = 9)]. **p<0.01 vs. wild-type mice. (**B, C**) Relationship between lumen area and total vessel area (**B**) or plaque area (**C**) in 16-, 32-, and 52-week-old apo E^−/−^ mice (black square) and wild-type mice (white circle).

The size of the atheromatous plaque area was positively correlated with the number of macrophages (Figure 3A), T-lymphocytes (Figure 3B), and microvessels (Figure 3C) distributed in the adventitial layer of the abdominal aorta in 16-, 32-, and 52-week-old apo E^−/−^ mice. In addition, the elastin content of the medial layer was negatively correlated with the numbers of macrophages (r = −0.55, p = 0.0102), T-lymphocytes (r = −0.61, p = 0.0040), and microvessels (r = −0.45, p = 0.0493). In contrast, collagen deposition was positively correlated with the number of macrophages (r = 0.53, p = 0.0099), T-lymphocytes (r = 0.54, p = 0.0092), and microvessels (r = 0.46, p = 0.0342). Figure 4A shows that the total vessel area was 2-fold larger in the abdominal aorta of 52-week-old apo E^−/−^ mice than in that of wild-type mice; however, the lumen area remained unchanged. The lumen area was positively correlated with the total vessel area (Figure 4B) but not with the plaque area (Figure 4C) at all ages of apo E^−/−^ and wild-type mice.

### Regional variations in IL-6, IL-10, and RANTES expression

**Figure 5:**
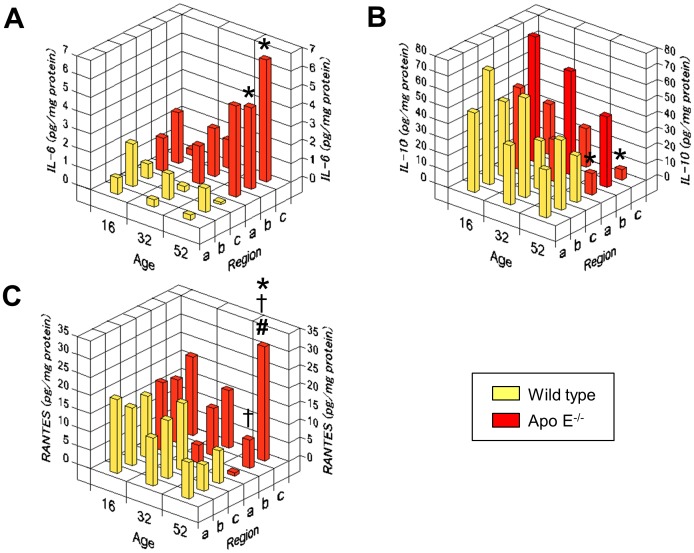
Impact of age on the region-specific concentrations of cytokine/chemokine in aortic tissue. Tissue concentrations of IL-6 (**A**), IL-10 (**B**), and RANTES (**C**) in the aortic arch (a), descending aorta (b), and abdominal aorta (c) of 16-, 32-, and 52-week-old apo E^−/−^ mice (red) and wild-type mice (yellow). The graphs present mean values of 3–4 samples. *p<0.05 vs. the respective region in 52-week-old wild-type mice; ^†^p<0.05 vs. aortic arch, and **^#^**p<0.05 vs. the descending aorta in apo E^−/−^ mice at 52 weeks.

Figure 5 compares the tissue concentrations of IL-6, IL-10, and RANTES in the aortic arch, descending aorta, and abdominal aorta of 16-, 32-, and 52-week-old apo E^−/−^ and wild-type mice. IL-6 concentrations tended to decrease with age in wild-type mice but dramatically increased with age in all aortic regions of apo E^−/−^ mice (Figure 5A). IL-10 concentrations decreased with age in both the groups (Figure 5B). In the aortic arch and abdominal aorta, values were significantly lower in apo E^−/−^ mice than in wild-type mice at 52 weeks. RANTES concentrations were similar in all aortic regions of wild-type mice and apo E^−/−^ mice at 16 weeks (Figure 5C). Subsequently, they tended to decrease with age in all aortic areas of wild-type mice. In apo E^−/−^ mice, the concentrations decreased in the aortic arch and descending aorta but increased in the abdominal aorta; these values were significantly higher than those in wild-type mice at 52 weeks.

### Immunolocalization of IL-6 and RANTES

**Figure 6:**
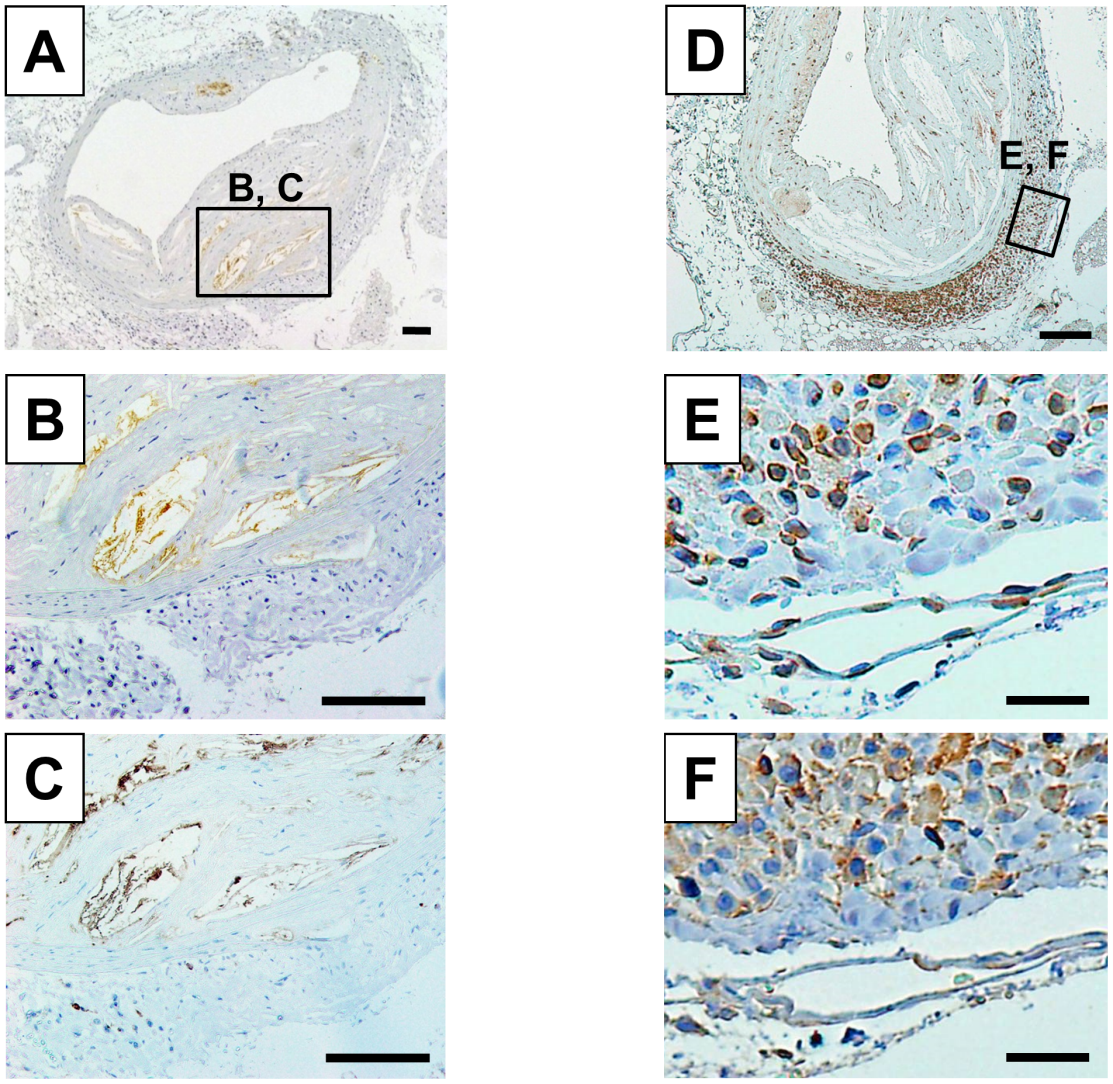
Localization of IL-6 and RANTES in the abdominal aorta of 52-week old apo E^−/−^ mice. Representative immunostainings for IL-6 (**A**) and RANTES (**D**) in the abdominal aorta of 52-week-old apo E^−/−^ mice. Square areas were magnified to verify the co-localization of IL-6- (**B**) and F4/80-positive macrophages (**C**) or the co-localization of RANTES- (**E**) and CD3-positive T-lymphocytes (**F**) in serial sections. Scale bar, 100 µm (**A–D**) and 20 µm (**E, F**).

In the abdominal aorta of 52-week-old apo E^−/−^ mice, IL-6 was immunodetected in the atheromatous plaque but not in the adventitial layer (Figure 6A). Serial-section analysis revealed that the IL-6-positive area (Figure 6B) was identical to F4/80-positive macrophages (Figure 6C). On the other hand, RANTES was detected more frequently in inflammatory cells of the adventitia than in the atheromatous plaque (Figure 6D). Serial-section analysis revealed that the RANTES-positive cells (Figure 6E) were mainly CD3-positive T-lymphocytes (Figure 6F).

## Discussion

Arterial remodeling is stimulated by physiological and pathological responses to vascular hemodynamics as well as immunological and biochemical factors [16], [18], [19], [20]. The present study provides evidence for an age-dependent and region-specific contribution of adventitial inflammation to the development of atherosclerosis.

Aging is the most important factor for the initiation and progression of atherosclerosis [21], [22]. Age-dependent structural changes in the aorta include intimal atheromatous formation and degeneration of the media along with a decrease in the elastin content and increase in collagen deposition [22], [23]. Human aorta exhibits age- and region-dependent increases in atherosclerosis, and the abdominal aorta acquires the largest intima/media ratio [24]. In the present study, the animal model of hyperlipidemia (apo E^−/−^ mice) developed more severe atherosclerosis in the aortic arch and abdominal aorta than in the descending aorta. The underlying mechanism by aging in the development of atherosclerosis may involve chronic low-grade inflammation, which modifies the gene expression of inflammatory cytokines and chemokines and senescence in the vasculature [25], [26], [27]. We noticed that the numbers of macrophages and T-lymphocytes in the adventitial layer of the abdominal aorta were higher in apo E^−/−^ mice than in wild-type mice at 52 weeks but not at 16 or 32 weeks. More importantly, the accumulation of these inflammatory cells in the adventitia coincided with a decrease in the elastin content and increase in collagen deposition in the aortic wall.

Circulating levels of many cytokines, chemokines, and adhesion molecules increase with age in humans [28], [29]. Furthermore, we demonstrated the existence of region-specific effects of age on cytokines and chemokines in aortic tissues of wild-type and hyperlipidemic mice. In wild type mice, aging was associated with stable or decreasing levels of IL-6, IL-10, and RANTES in the adventitia of the aortic arch, descending aorta, and abdominal aorta, from week 16 to week 52. In contrast, apo E^−/−^ mice exhibited an age-dependent increase in IL-6 levels, particularly in the descending and abdominal aorta. Furthermore, apo E^−/−^ mice exhibited a profound decrease in IL-10 levels with age, particularly in the aortic arch and abdominal aorta. Together, these data are consistent with the IL-6/IL-10 imbalance associated with the progression of atheroma formation [30]. Interestingly, compared with wild-type mice, RANTES levels showed a remarkable decrease in the aortic arch but a dramatic increase in the abdominal aorta of apo E^−/−^ mice with age. RANTES is expressed in various cell types, including monocytes/macrophages and T-lymphocytes [31], where it mediates the chemotactic activity of these cells during late-stage atherosclerosis [32], [33], [34]. In low-density lipoprotein receptor-deficient mice, RANTES was highly expressed in the atheroma of aortic root [35]. The present study suggests an active role for this chemokine in the adventitial layer as well. Thus, our data suggest that age-dependent and region-specific differences in cytokine/chemokine regulation in the adventitia contribute to atherogenesis.

The age-dependent formation of atheromatous plaque progressed similarly in the aortic arch and abdominal aorta. However, adventitial inflammation was strikingly more severe in the abdominal aorta. Why inflammatory cells accumulate in the adventitial layer of the abdominal aorta to a greater extent than that in the aortic arch and thoracic aorta remains unclear. Gräbner et al. [36] reported that lymphoid organs adjacent to the adventitia were more abundant in the abdominal aorta than in the other regions in aged-apo E^−/−^ mice, and that medial smooth muscle cells, activated by lymphotoxin β receptor signaling, facilitated the recruitment of lymphocytes into the aortic wall adventitia. Lymphotoxin β receptor signaling also induces the secretion of RANTES [37], [38], [39], suggesting that this chemokine amplifies the recruitment of inflammatory cells to the adventitia of the abdominal aorta. In addition, perivascular adipose tissue located close to the adventitia may affect immune responses in the aortic wall. As reported previously [40], [41], we observed that perivascular adipocytes were smaller in apo E^−/−^ mice than in wild-type mice. Nonetheless, perivascular adipocytes exhibited higher expression levels for inflammatory genes and markers of angiogenesis in the abdominal aorta than in the thoracic aorta [42].

Adventitial inflammation may represent an adaptive response to maintain blood supply to narrow vessels [43]. In this study, the lumen area was preserved despite the increase in the plaque size in the abdominal aorta of 52-week-old apo E^−/−^ mice. More importantly, the lumen area was correlated with the total vessel area but not with the plaque size. We reported earlier that adventitial and medial inflammation is associated with outward remodeling and activation of matrix metalloproteinase (MMP)-9 in the rabbit femoral artery [44]. Macrophages and T-lymphocytes express MMPs [45], [46], and these cells activate MMPs synergistically [47], [48]. Furthermore, RANTES stimulates MMP-9 transcription [49], leading to the degradation of elastin fibers in the abdominal aorta. In clinical settings, persistent inflammatory activation with angiogenesis may result in exaggerated arterial plaque instability and hemorrhage [50], [51], [52]. Alternatively, we speculate that the abdominal aorta is the preferential site of aneurysmal formation because adventitial inflammation may contribute to the excessive degradation of the extracellular matrix in the aortic wall [16].

In conclusion, this study describes the age-dependent and region-specific histological characteristics of adventitial inflammation during the progression of atherosclerosis in mice. Further studies are necessary to determine whether the imbalance in the cytokine/chemokine profile in the adventitial layer is a potential target for the attenuation of atherosclerosis and aneurysmal formation.
